# Ginkgo Biloba improves bone formation during fracture healing: an experimental study in rats

**DOI:** 10.1590/1413-785220172503156966

**Published:** 2017

**Authors:** Nizamettin Guzel, Emrah Sayit, Osman Aynaci, Servet Kerimoglu, Esin Yulug, Murat Topbas

**Affiliations:** 1 Samsun Education and Research Hospital, Department of Orthopedics and Traumatology, Samsun, Turkey; 2 Karadeniz Technical University Faculty of Medicine, Department of Orthopedics and Traumatology, Trabzon, Turkey; 3 Karadeniz Technical University Faculty of Medicine, Department of Histology and Embryology, Trabzon, Turkey; 4 Karadeniz Technical University Faculty of Medicine, Department of Public Health, Trabzon, Turkey

**Keywords:** Fracture healing, Ginkgo biloba, Rats

## Abstract

**OBJECTIVES:**

*Ginkgo biloba* extract (EGb 761) is a plant extract obtained from the leaves of the *G. biloba* tree. The aim of this study was to assess the histological and radiological effects of *G. biloba* extract on fracture healing in an experimental fracture model using rat femurs.

**METHODS:**

Forty-eight female Sprague-Dawley rats (weight: 195-252 g; age: 20 weeks) were used in the study. The rats were randomly divided into six groups (n=8). A transverse fracture was made in the middle of the right femur of each rat and fixed with a Kirschner wire. The *G. biloba* groups received 60 mg/kg oral *G. biloba* extract once daily. No medication was given to the control groups. On days 7, 21 and 35, both sets of femurs were evaluated radiologically and histopathologically.

**RESULTS:**

Histological evaluation revealed that the *G. biloba* groups had significant differences at 21 and 35 days (p<0.05). The *G. biloba* group showed a significant difference in terms of bone formation on day 21 when compared to the control group (p<0.05).

**CONCLUSIONS:**

This study indicated that the use of *G. biloba* extract accelerated fracture healing. Both radiological and histological differences were detected, but the histological differences were more remarkable. ***Level of Evidence I, High Quality Randomized Trial.***

## RESUMO

**OBJETIVOS:**

O extrato de Ginkgo biloba (EGb 761) é um extrato vegetal obtido das folhas da árvore Ginkgo biloba. O objetivo deste estudo foi avaliar os efeitos histológicos e radiológicos do extrato de Ginkgo biloba sobre a consolidação de fraturas em um modelo experimental de fratura em fêmures de rato.

**MÉTODOS:**

Foram utilizados 48 ratos Sprague-Dawley fêmeas (peso: 195-252 g, idade: 20 semanas). Os ratos foram divididos randomicamente em seis grupos (n = 8). Uma fratura transversal foi feita no meio do fêmur direito de cada rato e fixada com fio de Kirschner. Os grupos G. biloba receberam 60 mg/kg de G. biloba por via oral uma vez por dia. Não foi administrada nenhuma medicação aos grupos de controle. Nos dias 7, 21 e 35, ambos os fêmures foram avaliados radiológica e histopatologicamente.

**RESULTADOS:**

A avaliação histológica revelou que os grupos G. biloba apresentaram diferenças significativas aos 21 e 35 dias (p < 0,05). O grupo G. biloba mostrou uma diferença significativa em termos de formação óssea no dia 21 quando comparado com o grupo controle (p < 0,05).

**CONCLUSÕES:**

Este estudo indicou que o uso de extrato de G. biloba acelerou a consolidação de fraturas. As diferenças radiológicas e histológicas foram detectadas, mas as diferenças histológicas foram mais notáveis. ***Nível de Evidência I, Estudo Clínico Randomizado de Alta Qualidade.***

## INTRODUCTION

Many factors may affect the fracture healing process and blood supply at the fracture site has a direct effect.^1^ ^-^ ^3^ Blood vessels regenerate during the fracture healing process with the budding of existing blood vessels; if an adequate blood supply exists, the osteoblasts in the callus provide a matrix conductive to normal bone development. Oxygenation of the fracture site is one of the most important factors for fracture healing. Wu et al.^4^ reported that hyperbaric oxygen increased the proliferation and differentiation of osteoblasts. 

*Ginkgo biloba* extract (EGb 761) is a plant extract obtained from the leaves of the *Ginkgo biloba* tree, which has been proven to cause many metabolic effects such as vascular relaxation and increased blood volume, elimination of free radicals and reduction in secondary injury-induced tissue necrosis and cell apoptosis.^5^ ^,^ ^6^ In addition as a platelet-activating factor (PAF) antagonist and antioxidant, *G. biloba* improves blood circulation and helps prevent ischemic and reperfusion damage to tissue.^7^ Moreover, in patients with peripheral artery disease *G. biloba* has been shown to improve pain-free walking distance.^8^

In this study, we investigated the histological and radiological effects of *G. biloba* on fracture healing in an experimental rat model.

## MATERIALS AND METHODS

This study was approved by the institutional review board and ethics committee for animal experiments (KTU-hadyek/2010/41). Forty-eight female Sprague-Dawley rats (weight: 195-252 g; age: 20 weeks) were used; the rats were randomly divided into six groups (n= 8) after a 6-week compliance period. The first three groups were identified as Gb (*G. biloba*) 1, 2 and 3; the other three groups were classified as C (control) 1, 2 and 3. The Gb1 and C1 groups were followed for 7 days; Gb2 and C2 were followed for 21 days, while Gb3 and C3 were followed for 35 days. Patients in the Gb groups received 60 mg/kg oral *G. biloba* (EGb761) (Tebokan^(r)^ Forte, Abdiibrahim, Istanbul, Turkey) once daily. No medication was administered to the control groups.

### Surgical Procedure

**Figure 1:**
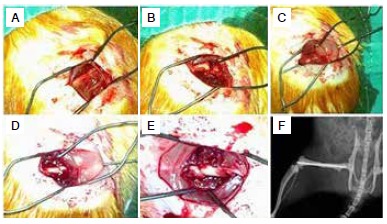
(A) An image of the femur after retraction of the soft tissues; (B) After the transverse fracture was obtained; (C) After placing an anterograde intramedullary K-wire from the fracture site to the knee; (D) Retrograde movement of the K-wire after reducing the fracture; (E) The last image of the fracture after pushing the K-wire inside the bone; (F) The postoperative roentgenogram of the fracture.

The rats fasted for 4 hours prior to surgery and received intraperitoneal injections of 5 mg/kg xylazine hydrochloride (Rompun^(r)^; Bayer Healthcare, Leverkusen, Germany) and 50 mg/kg ketamine hydrochloride (Ketalar^(r)^; Pfizer, Istanbul, Turkey) for anesthesia, with an additional 15 mg/kg ketamine hydrochloride administered if necessary. The rats were placed in the left lateral position and the surgical area was shaved. The skin of the right thigh was cleaned with 10% povidone iodine solution and a sterile field was created using appropriate covering before the surgery. A 2-cm lateral longitudinal incision was made at the right thigh. The fascia lata was split longitudinally and the vastus lateralis muscle was separated by blunt dissection from the fascia lata and the bone. (Figure 1A) Transverse holes were created using a 0.8-mm Kirschner wire and then a transverse fracture was gently created. (Figure 1B) A 1 mm diameter Kirschner wire was placed in an antegrade manner in the intramedullary canal towards the distal femoral condyles from the fracture site and then the wire was pulled distally. (Figure 1C) After fixation of the fracture, the wire was moved in a retrograde manner towards the greater trochanter. (Figure 1D) The wire stopped 2-3 mm before the greater trochanter and was cut distally with a wire cutter and pushed into the bone using another wire. After fixation, full reduction of the fracture site was achieved. (Figure 1E-F) The fascia and muscle were closed with absorbable sutures and the skin was stitched using nonabsorbable sutures. Lastly, the wound was cleaned with povidone iodine.

During the postoperative period, the rats were not prevented from bearing weight on the broken legs. No antibiotics were given before or after surgery. For postoperative analgesia, 4.5 mg/ml (150-250 mg/kg/day) paracetamol were added to the rats' water for 3 days after the surgery. The GB groups received 60 mg/kg oral *G. biloba* at the same time of day during the predetermined periods for each group. There were no complications related to the surgery or medication and all the rats survived.

**Figure 2:**
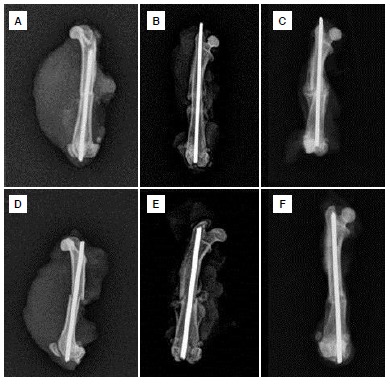
X-*ray* images of each group. (A) Gb1; (B) Gb2; (C) Gb3; (D) C1; (E) C2; (F) C3. (Gb: *Ginkgo biloba,* C: Control).

The rats in the Gb1 and C1 groups were sacrificed by cervical dislocation under general anesthesia on the seventh postoperative day. The Gb2 and C2 rats were sacrificed 21 days following surgery and the Gb3 and C3 groups were sacrificed on the 35^th^ postoperative day. The right femurs of all rats were disarticulated from the hip and the knee. The soft tissues were stripped for radiological evaluation and anteroposterior and lateral radiographs were taken of the right femurs. (Figure 2A-F) All X-rays were assessed using the Lane-Sandhu radiological scoring system,^9^ and were scored by a different orthopedic surgeon who was unaware of the study and the groups.

After the X-rays were obtained, the samples were placed in different containers numbered separately for the histologic evaluation which were filled with a 10% neutral formaldehyde solution. A 10% formaldehyde solution with formic acid was used for decalcification of the fixated tissues. Paraffin was cut into 5-micrometer-thick blocks using a fully automatic microtome (Leica RM2255, Japan). Hematoxylin and eosin staining was used to show the overall structure and Masson's trichrome was employed to distinguish the connective tissue.

**Figure 3:**
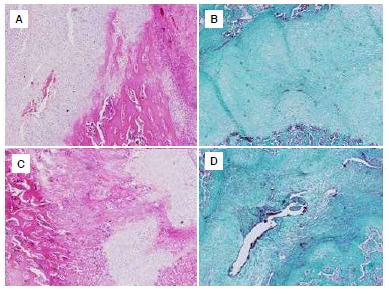
Abundant cartilaginous tissue was seen in the control group on day 21; (A) Hematoxylin and eosin staining x20; (B) Masson's trichrome x10). Abundant cartilaginous tissue and immature bone formation; (C) Hematoxylin and eosin staining x10) and immature bone spicules and red bone marrow; (D) Masson's trichrome x10) were seen in the Ginkgo biloba group at day 21.

**Figure 4:**
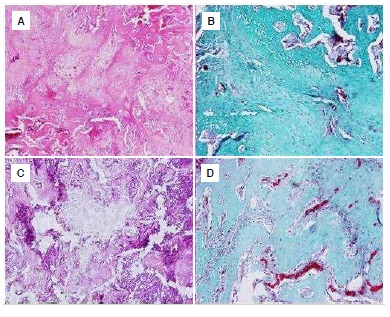
(A) A fracture line containing equal amounts of immature bone and cartilage tissue was observed in the control group on day 35 (Hematoxylin and eosin x10). (B) Equal amounts of cartilage and immature bone containing red bone marrow were seen in the control group on day 35 (Trichrome's Masson x20). (C) A fracture line containing abundant immature bone tissue and a small amount of cartilaginous tissue was seen in the *Ginkgo biloba* group on day 35 (Hematoxylin and eosin x20). (D) A fracture line containing abundant bone spicules, red bone marrow and a small amount of cartilaginous tissue was seen in the *Ginkgo biloba* group on day 35 (Trichrome's Masson x20).

The sample preparations were evaluated by an experienced independent histologist under a light microscope (Olympus BX51-Japan) and were photographed with a digital camera under the light microscope. (Figure 3A-D) The histological scoring system described by Huo et al.^10^ was used for to evaluate the preparations. (Figure 4A-D) 

### Statistical analyses

The Statistical Package for the Social Sciences (SPSS, version 20 for Windows; SPSS Inc., Chicago, Illinois, USA) was used for all statistical analyses. The Kolmogorov-Smirnov test was performed to determine if the data were normally distributed. The Mann-Whitney U test was employed to compare the radiological and histopathological evaluations between the two groups. The data were summarized as median (minimum-maximum) values. A p-value <0.05 was considered statistically significant.

## RESULTS

The X-ray scoring subgroups were evaluated separately. No statistically significant differences were found between the *G. biloba* and the control groups on day 7 or day 35 in terms of bone formation (p >0.05), whereas a significant difference between the groups was seen on day 21 (p<0.05). (Table 1) There was no statistically significant difference between groups in terms of union, remodeling, or the total radiological score (p>0.05). (Tables 2, ^3^ and ^4^)

**Table 1:** The median (minimum-maximum) radiological scores of all groups according to bone formation.

	Bone formation		
	Day 7	Day 21	Day 35
Gb	3(2-4)	3(3-4)	4(1-4)
C	2,5(1-3)	3(2-3)	3(1-4)
p	0,076	0,018*	0,534

Gb: Ginkgo biloba. C: Control. * indicates statistical significance.

**Table 2:** The median (minimum-maximum) radiological scores of all groups according to the union.

	Union		
	Day 7	Day 21	Day 35
Gb	2(0-4)	2(2-4)	4(0-4)
C	2(0-2)	2(2-2)	2(0-4)
p	0,199	0,143	0,174

Gb: Ginkgo biloba. C: Control.

**Table 3:** The median (minimum-maximum) radiological scores of all groups according to the remodeling.

	Remodeling		
	Day 7	Day 21	Day 35
Gb	2(0-4)	2(2-4)	4(0-4)
C	2(0-2)	2(2-4)	2(0-4)
p	0,464	0,535	0,174

Gb: Ginkgo biloba. C: Control.

**Table 4:** The median (minimum-maximum) total radiological scores of all groups.

	Total radiological score		
	Day 7	Day 21	Day 35
Gb	7(2-12)	7(7-12)	12(1-12)
C	6,5(1-7)	7(6-9)	7(1-12)
p	0,124	0,063	0,377

Gb: Ginkgo biloba. C: Control.

Following the histopathological evaluation, no statistically significant difference was found between the groups on day 7 (p>0.05); however, there were statistically significant differences between the groups on day 21 and 35 (p<0.05). (Table 5)

**Table 5:** The median (minimum-maximum) histopathological scores of all groups.

	Histopathological score		
	Day 7	Day 21	Day 35
Gb	2(1-2)	6(5-7)	8,5(7-10)
C	2(1-2)	5(4-6)	6(6-7)
p	0,535	0,009*	0,001*

Gb: Ginkgo biloba. C: Control. * indicates statistical significance.

## DISCUSSION

The effects of different drugs or substances have been investigated by many researchers.^11^ ^,^ ^12^ However, there have been no studies to date that have explored the effects of *G. biloba* on fracture healing. 

*G. biloba* is a well-known vasoregulatory agent that reduces blood viscosity and increases blood flow.^13^ Ginkgolide B, one of the components of *G. biloba*, has been particularly reported to act as an antiaggregant by antagonizing PAF. In this way, *G. biloba* decreases neutrophil degranulation and the production of oxygen radicals which stimulate platelet aggregation.^14^ *G. biloba* has been reported to produce a strong vasorelaxant and antiaggregant effect with its superoxide anion cleansing action, which prolongs the half-life of endothelium-derived relaxing factor (EDRF).^15^

*G. biloba* has also been used to prevent neurotoxic conditions by inhibiting c-Fos translocation, which causes glutamate-induced up-regulation of tissue plasminogen activator.^16^ Yan et al. showed that administering EGb761 during the acute phase following spinal cord injury significantly reduced secondary injury-induced tissue necrosis and cell apoptosis and improved functional performance in rats.^6^

*G. biloba* extract has been used for many clinical conditions such as concentration and memory problems in elderly patients, anxiety and depressive diseases, dizziness and tinnitus.^17^ ^,^ ^18^ Treatment with *G. biloba* produces significant differences in cerebral insufficiency symptoms.^19^ However, there have been no clinical or experimental studies that have investigated the effects of *G. biloba* on fracture healing. Our experimental fracture model revealed that this compound made significant differences in fracture healing which were demonstrated both radiologically and histopathologically in this study. Histologically, those effects were more distinctive on the 21^st^ and 35^th^ days following the fracture. However, radiological scoring revealed that a significant difference in bone formation only occurred on day 21.

Blood supply to the fracture area is important in fracture healing; *G. biloba* extract increases oxygen uptake into cells by increasing blood circulation in tissues and allows the removal of toxins from the environment.^20^ This effect occurs because *G. biloba* is a PAF antagonist, has antioxidant properties, removes free radicals from the environment, provides vascular relaxation and increases the blood supply and oxygenation of the tissues by reducing blood viscosity. Increased blood supply to the fracture site leads to higher concentrations of mediators and cytokines, which aid in the fracture healing process.

In this study, although the radiological scores of the *G. biloba* group for bone formation were higher on day 7 and day 35, there was only a significant difference on day 21 between the two groups. This may occur because the effects of revascularization are more important between days 7 and 35 for new bone formation. Union and remodeling take place after bone formation, so the radiological scores of both groups were nearly the same according to union and remodeling. Scores improved on day 35, but were not statistically significant. These findings support the suspicion that *G. biloba* causes accelerated bone formation but has no significant effects on final union or remodeling.

According to the histopathological scores, there were significant differences between the *G. biloba* and control groups on days 21 and 35. Fibrous and cartilaginous tissues formed during the early stages of bone healing, leading to the subsequent development of both immature and mature bone. The effects of *G. biloba* on bone formation were significant during the late phases of healing. 

One limitation of our study was the lack of different dosages of *G. biloba*. Further studies with different dosages are required to obtain more information about the effects of *G. biloba*.

## CONCLUSION

This study showed that use of *Ginkgo biloba* accelerated bone formation and fracture healing. Both radiological and histological differences were detected, but the histological differences were more notable.
